# Westminster Hospital

**Published:** 1829-04-01

**Authors:** 


### WESTMINSTER HOSPITAL.

There have been several operations and cases of great interest in this hospital of late.

HERNIA.—James Bentley, aged 30, was admitted, October 15, at half-past ten, with strangulated scrotal hernia, of a large size. He had been bled previously to 20 ounces, and was placed in the warm bath. Mr. Guthrie arriving at half-past 12, examined the patient, and finding the strangulation had continued for 20 hours, and that there was great pain at the umbilicus and in the hernia, on the slightest pressure, proceeded to perform the operation.

An incision was made from the upper edge of the swelling, to the extent of three inches and a half. The cellular texture and fasciæ, which latter were not distinct, were divided, and a very thin peritoneal sac exposed: a small opening being made towards the lower part of it, the omentum appeared; and on the sac being slit up, a little fluid escaped, and the contents were fully brought into view, being a very large portion of omentum and colon, amounting to half a hatfull, the omentum being thin, membranous, of a grey colour, with its vessels ramifying on it in a very distinct manner. The colon inflated and inflamed. The fascia of the external oblique having been slit up, the stricture was seen to be at the inner ring, which was divided upwards, so as easily to admit the fore-finger, which could be passed either before or behind the intestine into the abdomen. Portions of intestine and omentum were drawn down, in order to facilitate their reduction, but the efforts of the patient, who could not be held steadily, or kept from calling out, together with the weight of so large a quantity of intestine and omentum, caused each part to protrude again as fast as it was returned. Mr. Guthrie, with that coolness and presence of mind for which he is so remarkable in all operations, seeing the cause of the difficulty, requested Mr. Lynn to return the intestine, whilst he had the patient's body bent forward, and collected and supported all the protruded parts with both hands, so as to take off their weight, when the whole was returned without the slightest difficulty, and Mr. Guthrie placed his three fore-fingers in the incision through the stricture, to shew the size of the opening. The wound was closed by a suture, sticking-plaster, and bandage. An enema was directed to be given and repeated, and sixteen ounces of blood to be drawn from the arm within the hour, if no signs should occur forbidding it. Before Mr. Guthrie left the hospital, he found the pain in the abdomen had returned, he therefore directed 20 ounces of blood to be immediately drawn from the arm. Half-past four.—Mr. Guthrie visited the patient, and directed 12 ozs. more blood to be drawn, which almost immediately lowered the pulse. Half-past eight.—Mr. Guthrie, finding the patient had had four evacuations, and that the symptoms of inflammation were fast disappearing, ordered:—℞. Hydrarg. submur. gr. vj. Ext. opii, gr. jss. statim. Ol. ricini, ℥j. mane.

16th. Mr. Guthrie visited the patient at 8 o'clock, who had passed a tolerable night; but there being some pain near the wound, he directed 20 leeches to be applied to the abdomen over the spot; pulse 108, neither hard nor full. Half-past three.—The leeches bled well, and he is much relieved—has had four motions since morning: diet, weak tea only. ℞. Ol. Ricini, ʒij. sextis horis.

17th. Going on well; pulse 94—bowels well open.

18th. Free from symptoms. Ol. ricini, ℥ss. statim.

19th. Sleeps well, and says his appetite is good.

20th. The wound is uniting. To take the common house purgative medicine every morning it may be required.

31st. Complains of a swelling near the umbilicus, on the same side as the hernia, which, on examination, is painful, and appears to be inflammation of the cellular texture, below the external oblique muscle. To have 20 leeches applied and repeated, with warm evaporating poultices. ℞. Pulv. jalap. c. ʒj. statim.

Nov. 1st. Is better, but the swelling continues—leeches repeated.

20th. The swelling, which was never accompanied by external inflammation, at last pointed close to the umbilicus, and an opening being made, a tea-spoonful of matter was discharged, and the wound healed in a day or two.

Dec. 1st. The hardness which remained after the little opening closed, suddenly descended, with some external inflammation, to the line of incision made in the operation, and an abscess soon pointed, which was opened, and a table-spoonful of matter was discharged.

Dec. 7th. Discharged cured, being furnished with a truss.

Mr. White operated in another case of a similar description, and on the same side, a few days afterwards, in the person of an old man of 74. The hernia, a very large scrotal one, had been strangulated three days when he was brought to the hospital. The man was very weak and exhausted, the danger imminent. Mr. White performed the operation the moment he arrived. On opening the sac, the intestine was found of a black colour and nearly in a state of mortification, and in considerable quantity, and a great deal of very fetid fluid escaped. The same difficulty was experienced in returning the intestine as in the former case. Mr. White enlarged the opening; still the intestine could not be returned, until, in a manner similar to that in the case of Bentley, the intestine was raised and supported, when Mr. White returned it without difficulty. The man was put to bed, but died about an hour afterwards.

In a clinical lecture delivered on the subject of these two cases, Mr. Guthrie particularly impressed on the students the importance of the fact, that when a large quantity of intestine and omentum were protruded, it was hardly possible to return them with only a moderately-sized incision of the stricture, unless the parts were supported, so as to take off their weight, and the action of the abdominal muscles was quieted. Of which these two cases gave ample proof. He had had, he said, a similar case some years back, which terminated most favourably. He was sent for during lecture to the patient, who lived in Tottenham Court Road, and, finding the symptoms urgent, he operated immediately; the quantity of small intestine protruded was so great, as to resemble a link of sausages hanging up in a shop. The stricture being divided, he attempted to return a part of the gut: finding it would not go up, he drew a portion down from within the abdomen; but still the difficulty remained. After several ineffectual trials, even to restore the pieces he had drawn down, he at last thought of supporting the whole above the level of the wound, when the intestine was readily returned, piece by piece, in succession. Mr. Guthrie desired the students would observe, that if so much difficulty existed when the stricture was divided, they must expect it to be infinitely greater before any operation was performed; and therefore it was, that very large scrotal herniæ usually required an operation for the relief of the patient, more particularly if they had been previously reducible. It was from this knowledge, gained by experience, he had been led to operate in the case of Bentley, after only an examination of the state of the part, and without trying any other means of relief.

This case was, he said, infinitely more valuable on another account. It shewed the necessity of an attentive and well directed medical treatment after the operation. Without it, the operation would have been useless. It not only consisted in the abstraction of blood, but in the refraining from administering purgatives, until the inflammatory action was subdued, and then only in such quantities as would act in a very gentle manner. There was no error in medical surgery so great as that, which many surgeons fell into, and which was even recommended from high authority, viz. of having recourse to severe purgation after an operation for hernia. Repeated glysters and gentle purgatives, combined even with opium, were greatly to be preferred, and it was only when all fear of inflammation seemed to have passed away, that others more active might be had recourse to, and then even, he said, they might be very injurious, and instanced a case, in which too severe a purgative having been accidentally given, brought on from the wound a fecal discharge, which happily, however, subsided, and the patient recovered. In the case operated upon by Mr. White, the mischief had gone too far, both locally and generally, to be arrested by the operation, but it was very satisfactory to observe, how much the black colour of the intestine had changed to a brown, and even, in some parts, to a red colour, after the stricture had been taken off.

### AMPUTATION.

There have been three amputations, one of the thigh and two of the leg.

James King, aged 35, admitted Sept. 3d, with a diseased ankle-joint, under which he had laboured for four years past. The amputation was performed by Mr. Harding, on the 29th November, by the double flap operation, the principle one being formed from the under part; two sutures were inserted on each side of the tibia to prevent the application of sticking plaster over it; little blood was lost during the operation, which was done with great precision and dexterity. On the 30th, the pulse being full and quick, with pain in the side, twelve ounces of blood were directed to be taken from the arm, which was cupped and buffy. Haustus catharticus.

2d December. The pulse continuing quick, and some uneasiness in the stump, with a painful sensation of feeling in the toes, V.S. ad. ℥xij. The stump has been kept wet and cold. Ol. ricini, ℥ss. The dressings were partially removed yesterday afternoon.

3d. Complains of pain in the stump, but that in the side is gone; bowels open; has passed a good night; tongue clean; pulse 100. The union of the edges of the incision seems complete, except where the ligatures were brought out.

4th. Passed a good night; bowels open; pulse 98; feels pain which he refers to the great toe.

6th. Says he did not sleep from the pain in the toe, but is much easier this morning. Bowels regular; tongue clean; pulse 100. The pain on the outside of the tibia.

7th. After the stump had been dressed this morning it began to bleed, and about 8oz. were lost before compression could be made on the artery. He had no pain before it came on, but there was a good deal of twitching and jerking of the stump. Pulse 120, and feeble.

The stump was examined, when the outer part between the tibia and fibula appeared in a sloughy state, and from this part it bled generally; an equal compression being made upon it, and the part bandaged, the hæmorrhage was suppressed. A mild purgative given; some wine; his dinner a mutton-chop.

8th. Is tolerably free from pain; no return of the hæmorrhage; passed a good night; pulse 136.

In the afternoon the hæmorrhage returned. Mr. White finding no particular vessels bleeding, and that a ligature cut through the part, removed half an inch of the surface to reach a sound part, when the hæmorrhage was effectually stopped. The man gradually sunk, and died in the evening.

On examination, the whole of the stump appeared perfectly healthy, with the exception of the single sloughing spot alluded to. The anterior tibial artery and veins were sound. The man was in a weak state prior to the operation, from long-continued disease.

Moses Barnes, aged 49, was brought to the hospital, December 1st, the right leg having been torn off in consequence of the foot having caught in the iron chain trace of a cart-horse who was plunging violently at the time. The tibia projected several inches beyond the muscles, which were torn very irregularly. The man suffered great pain at the time, which continued. Mr. White, on his arrival, which was on being sent for, removed the remaining portions of the leg, by the flap operation about three inches below the tuberosity.

2d. The patient passed a restless and sleepless night, complaining of pain and twitching of the limb. He is this morning easier, but is evidently much exhausted. Pulse 132, weak and not regular. Tongue dry in the middle, the eyes clear.

3d. Died in the night, wine, opium,

&c. having been given him without the slightest effect. Mr. White remained himself with him until 2 o'clock.

The patient was a short, thick, strong, and rather corpulent man. Mr. Guthrie, who happened to be in the hospital before Mr. White arrived, desired the students to attend particularly to the case, and to the appearance of the man, the impression on his mind, being that he was a bad subject for the accident, and that the termination would not be fortunate.

Christopher Stout, aged 36. Thigh amputated for disease of the knee-joint, (ulceration of the cartilages,) Dec. 6th, 1828. The operation was performed by Mr. White, with facility and dispatch, and the patient is nearly well.

Mr. Guthrie took the opportunity after the amputation of shewing to the students the stump of a man, John Bartholomew, whose leg he had amputated 12 years ago. The tibia had been sawn through at the tuberosity, and the head of the fibula had been removed. A sufficient portion of tibia had been left to rest upon. The consequence was, that this man could walk four miles an hour on his wooden leg with ease, and was in the habit of carrying one hundred weight on his head from Covent Garden into Westminster, near the hospital, twice every day. Mr. Guthrie said, that by leaving the knee-joint, and the attachment of the inner hamstring, the straight progressive motion of the limb was preserved, and which was always lost when the knee was removed. The patient waddled, and had great difficulty in preserving his equilibrium. The only danger in the removal of the fibula was, that the head of it in perhaps one case in 20, entered into the composition of the knee-joint, which circumstance might in general be ascertained by a careful examination of the fibula previous to the operation.

